# Theragnostic chromosomal rearrangements in treatment‐naive pancreatic ductal adenocarcinomas obtained via endoscopic ultrasound

**DOI:** 10.1111/jcmm.16381

**Published:** 2021-03-11

**Authors:** Stephen J. Murphy, Michael J. Levy, James B. Smadbeck, Giannoula Karagouga, Alexa F. McCune, Faye R. Harris, Julia B. Udell, Sarah H. Johnson, Sarah E. Kerr, John C. Cheville, Benjamin R. Kipp, George Vasmatzis, Ferga C. Gleeson

**Affiliations:** ^1^ Biomarker Discovery Laboratory Centre for Individualized Medicine Mayo Clinic Rochester MN USA; ^2^ Division of Gastroenterology & Hepatology Mayo Clinic Rochester MN USA; ^3^ Department of Anatomic Pathology Mayo Clinic Rochester MN USA

**Keywords:** chromoanagenesis, endoscopic ultrasound guided biopsies, genomic rearrangements, mate pair sequencing, neoantigens, pancreatic ductal adenocarcinoma, structural variance analysis

## Abstract

A crucial mutational mechanism in malignancy is structural variation, in which chromosomal rearrangements alter gene functions that drive cancer progression. Herein, the presence and pattern of structural variations were investigated in twelve prospectively acquired treatment‐naïve pancreatic cancers specimens obtained via endoscopic ultrasound (EUS). In many patients, this diagnostic biopsy procedure and specimen is the only opportunity to identify somatic clinically relevant actionable alterations that may impact their care and outcome. Specialized mate pair sequencing (MPseq) provided genome‐wide structural variance analysis (SVA) with a view to identifying prognostic markers and possible therapeutic targets. MPseq was successfully performed on all specimens, identifying highly rearranged genomes with complete SVA on all specimens with > 20% tumour content. SVA identified chimeric fusion proteins and potentially immunogenic readthrough transcripts, change of function truncations, gains and losses of key genes linked to tumour progression. Complex localized rearrangements, termed chromoanagenesis, with broad pattern heterogeneity were observed in 10 (83%) specimens, impacting multiple genes with diverse cellular functions that could influence theragnostic evaluation and responsiveness to immunotherapy regimens. This study indicates that genome‐wide MPseq can be successfully performed on very limited clinically EUS obtained specimens for chromosomal rearrangement detection and potential theragnostic targets.

## 1 INTRODUCTION

Pancreatic ductal adenocarcinoma (PDAC) is an aggressive, typically densely fibrotic tumour, that is predicted to become the cancer with highest mortality by 2025, with an overall five‐year survival of < 10%.^1^, ^2^ Current clinical care guidelines endorse both germline and somatic testing as a form of universal genetic testing to identify actionable targets. PDAC is thought to evolve slowly, presenting at a late stage with a poor prognosis, and follows a sequence of common genetic alterations: *KRAS* → *CDKN2A* → *TP53 / SMAD4*.^3^, ^4^ Although the frequencies of these mutations are reported at 93%, 78%, 34% and 32%, respectively,^3^, ^4^, ^5^, ^6^, ^7^, ^8^, ^9^ the absence of FDA‐approved targeted therapy aimed at these common alterations has limited genomic‐driven individualized therapy. A retrospective analysis of 1,856 PDAC patients enrolled in the Know Your Tumor programme demonstrated that 26% had actionable molecular alterations, but overall, only 4% of all tested patients received a molecularly matched therapy.^8^

Routine somatic mutational analysis of PDAC has focussed upon comprehensive cancer gene panels or whole exome sequencing for single‐nucleotide variants (SNV) and small insertions and deletions (InDels).^4^, ^5^, ^6^, ^7^, ^8^, ^9^ However, the high levels of aneuploidy and large genomic rearrangements greatly impact gene expression and their presence has highlighted the need for more integrated genomics to include specialized structural variance analysis (SVA), transcriptomics (RNAseq) and epigenomics to fully evaluate potential therapeutic targets. Studies considering SVA, in addition to SNVs, have increased the observed prevalence of *SMAD4, CDKN2A* and *TP53* deleted cases through more specialized detection of larger deletions and breakpoint junctions, hitting important tumour suppressor genes.^10^

Genome instability is a hallmark of cancer and a progressive mechanism driving cancer progression. Large genomic rearrangements generate discordant mapping DNA junctions that frequently impact the expression of important regulatory genes in many cancers including PDAC.^10^ Newer generations of DNA sequencing technologies have additionally exemplified a newer class of genomic alteration termed chromoanagenesis, which involves tens to hundreds of genomic rearrangements that appear to be derived from a single catastrophic event, rather than many incremental steps.^11^, ^12^, ^13^ This drives the concept that a cancer genome can evolve in a rapid burst. Up to 27% of all human cancers and > 60% of PDAC contain chromoanagenetic events, affecting one or more chromosomes.^11^, ^12^, ^13^ Chromoanagenesis has been associated with tumour aggression and poor patient survival in several malignancies, including PDAC, but the outcome is less predictive in other cancers.^12^ The impact of chromoanagenesis on altered gene structures is thought to generate an increased frequency of expressed neo‐peptides potentially influencing clinical responses to immunotherapy.^2^, ^13^

Endoscopic ultrasound (EUS)‐guided biopsy specimens have the unique potential to provide an early snapshot of the tumour mutational landscape.^3^, ^7^, ^14^, ^15^, ^16^ Concerns arise, however, that interpretation of genomics data from image guided biopsies may be limited by quality control criteria. Recent multicentre studies of radiology‐guided percutaneous needle biopsy specimens noted that only 74% of biopsies were of satisfactory quality for next‐generation sequencing (NGS) limited gene panels.^14^, ^15^, ^16^ The greatest limitation was the inadequate specimen size and tumour content, with 19% of more than 400 broad spectrum tumour core biopsy specimens reporting tumour percentages of ≤ 30%.^14^ As such, the National Cancer Institute suggests that specimens with > 50% viable tumour are optimal and those ranging from 25%‐50% are acceptable for molecular testing. However, the ability to identify relevant genomic alterations decreases with decreasing tumour content. Given the cited challenges and requirements, we sought to assess 1. the feasibility of complete SVA, 2. prevalence and pattern of chromoanagenesis and 3. identifiable possible prognostic and therapeutic targets in treatment‐naïve pancreas cancer EUS biopsy specimens, utilizing a whole genome‐based technique called Mate Pair sequencing (MPseq).^10^, ^13^, ^17^, ^18^, ^19^, ^20^

## 2 MATERIALS AND METHODS

### 2.1 Patient selection

All patients were consented under Mayo Clinic Institutional Research Board (IRB) guidelines (IRB # 14‐009347): the EUS cohort (n = 12: PDAC = 11 and pancreas neuroendocrine tumour (pNET) =1 for comparative purposes) for MPseq was comprised of 6 men and 6 women [68 years (IQR 64.5‐75), Ca19‐9 at diagnosis: 477 U/ml (IQR 21‐1,630.5)], 8 (66%) of whom had a solid pancreas head lesion with an EUS long axis of 34mm (IQR 26.5‐44.5mm) and were followed for 42.9 (IQR 10.6‐50.5) months. Clinical disease stage ranged from resectable (n = 1; 8.3%) to locally advanced (n = 6; 50%) or metastatic disease (n = 5; 41.7%). The spectrum of AJCC disease stage based upon EUS findings was IB (n = 1; 8.3%); IIA (n = 1; 8.3%); III (n = 5; 41.7%); and IV (n = 5; 41.7%). Patients initially elected for either no therapy (n = 3; 25%); neoadjuvant therapy (n = 6; 50%); or palliative therapy (n = 3; 25%). Six patients (50%) proceeded to oncologic surgery, 3 of whom represented a complete pathologic response to neoadjuvant therapy (ypT0N0). Disease progression was observed in 5 (41.7%) patients, each with hepatic metastasis. Overall observed mortality was n = 4 (33.3%) at 10.1 (IQR 5.4‐17.2) months following EUS diagnosis. Patient clinical demographics are summarized in Table S1.

### 2.2 Biopsy procedure and sample processing

A 22G EUS fine‐needle aspiration (FNA) (Wilson Cook, Winston‐Salem, NC) or fine‐needle biopsy (FNB) needle (SharkCore FNB needle Medtronic Corp., Boston, MA) was used to diagnose and stage a treatment‐naïve solid pancreatic mass with the aid of rapid on‐site evaluation. Once diagnostic material was confirmed, a dedicated pass was performed and snap frozen and stored at −80°C for subsequent DNA/RNA extraction. Cytologic smears were assessed for molecular testing adequacy metrics to include cellularity ^15^ DNA and RNA were co‐extracted using the Qiagen ALLprep kit (#80284) according to manufacturer's instructions and quantitated using the Qubit fluorometer (ThermoFisher #Q33327). Sanger sequencing was utilized to determine the G12 KRAS mutation status of each case from PCR amplicons as described previously.^9^

### 2.3 MPseq protocol

The MPseq large fragment (2‐5kb) tiling protocol was used to detail genomic structural variants, including copy number variations (CNV) and discordant mapping genomic junctions.^17^, ^18^, ^19^, ^20^ MPseq libraries were assembled from 1µg of DNA using the Nextera Mate‐Pair Kit (Illumina, CA, FC‐132‐1001) following the manufacturer's instructions. Libraries were sequenced on the Illumina HiSeq4000 platform at a depth of 4 libraries per lane. Sequencing statistics data are presented in Table S2.

The binary indexing mapping algorithm (BIMA) simultaneously maps both fragment reads to the GRCh38 reference genome.^20^ Structural variants were detected using SVAtools, a suite of algorithms developed by the Mayo Clinic Biomarker Discovery Laboratory.^17^ SVAtools specifically detects discordant fragments supporting a common junction (supporting fragments) with powerful masks and filters to remove false‐positive junctions. CNV detection is performed using the read count of concordant fragments within non‐overlapping bins.^18^ A sliding window statistical method is used to determine likely copy number edges from read depth, as well as using breakpoint locations determined in the junction detection stage. The normalized read depth (NRD) for a region was calculated as two times the read depth within a region divided by the expected read depth for normal diploid level for the sample. Chromosomal copy levels and discordant mapping junctions were visualized on interactive software for genome plots.^19^

### 2.4 Determinations of tumour percentage

The tumour percentage of a sample is determined from the proportion of tumour mapping fragments within the MPseq data, determined through copy number analysis. The CNV detect algorithm determines the read distribution frequency for losses (0N, 1N), normal diploid (2N) and gains (3N, 4N, 5N, etc). For 100% tumour, these peaks would be expected to locate at the integer values; 0, 1, 2, 3, 4, 5, etc; however, for < 100% tumour the peaks are shifted proportionally to the contaminating 2N normal tissue genomes. The positioning of the 1N and 3N peaks with respect to the 2N peak enables accurate percentage tumour calculations utilizing the formulas: %tumour = [(2N‐1N)/(2N/2)]*100% or [(3N‐2N)/(2N/2)]*100%.

### 2.5 RNAseq protocol

RNAseq libraries were prepared on a subset of PDAC specimens (n = 4), where 200ng total RNA (RIN > 7) was available, using the Illumina TruSeq protocol and multiplexed on a HiSeq4000 single lane. The R/Bioconductor environment (https://www.bioconductor.org/) was used for transcriptome analysis. Paired end sequence fragments were aligned by the Tophat aligner using the hg38 reference genome and the ensemble annotation database and the htseq program calculated expression levels of genes in each sample. The edgeR package was then used to normalize all gene expression levels. Discordant mapping RNA transcripts (Fusions and Neo‐peptides) were reported with > 3 supporting fragments or any number of supporting fragments for transcripts adjacent to junctions reported in MPseq.

## 3 RESULTS

### 3.1 Whole genome mate pair sequencing of EUS fine‐needle specimens

**FIGURE 1:**
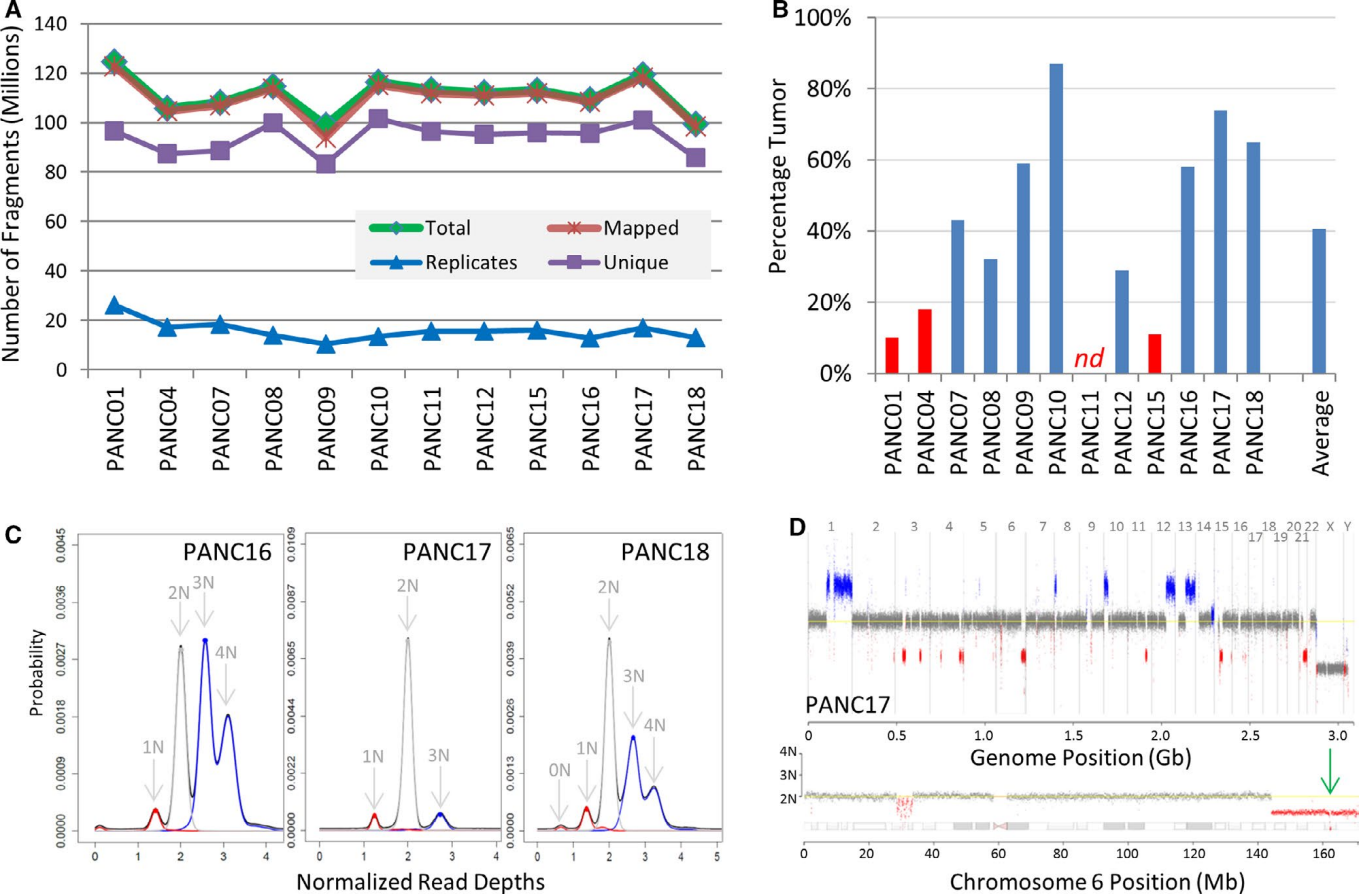
MPseq SVA. A, MPseq statistics. Number of total, mapped, unique and replicate fragments per sample. B, Predicted percentage tumour for each case and average. Cases with < 20% tumour are indicated in red, with one case not determinable, nd. C, Normalized read‐depth plots for PANC16, PANC17 and PANC18. The normalized read depths are presented on the x‐axis with probability coverages on the y‐axis. The predicted integer ploidy levels for coverage levels indicated with arrows. D, Genome linear plot for case PANC17. Read coverage in 30kb window sizes across genome displayed horizontally and sequentially for chromosomes 1‐22, X and Y. Normal diploid 2N level presented by grey dots. Gains and losses indicated by blue and red dots, respectively. Lower panel indicates coverage across exemplar chromosome 6, with an additional homozygous loss within PARK2 indicated by a green arrow at the 6q26 locus."

Twelve pancreatic cancer patients were recruited under full IRB for a research pass EUS‐guided biopsy as detailed in methods. Eleven of these patients were subsequently diagnosed with PDAC and one patient with a pancreatic neuroendocrine tumour (pNET) with patient clinical demographics summarized in Table S1. DNA was extracted, and MPseq was successfully run on all (n = 12) EUS‐guided biopsy specimens. An average of 111 million fragments was yielded per sample, with approximately 99% mapping efficiency and 14% replicate fragments (Figure 1A, Table S2). Biallelic bridged coverage, considering the span of the larger fragments, averaged 68X (54‐82X) and was more than adequate for effective SVA.^17^ Base coverage averaged 8X, which precludes point mutation calling through MPseq. Tumour percentages were determined for each case (Figure 1B) from the positioning of 1N and 3N copy levels relative to the normal diploid 2N in the normalized read‐depth (NRD) distribution curves ^18^ (Figure 1C). The 1N, 2N and 3N peaks would lie at 1, 2 and 3, respectively for a 100% tumour specimen. The levels of normal tissue (2N) contained within a specimen shifts the positioning of the NRD peaks to predictable values, allowing accurate determinations of tumour percentage. A mean tumour percentage of 41% was observed for the 12 specimens studied. (Figure 1B and Table 1).

**TABLE 1:** Summary of case genomics

**Case**	**Type**	**EUS Needle**	**Tumour %**	**Gene Copy Levels^**			**SVA Quality**	**Complex Rearrangements**		**Sanger *KRAS***
				***p16***	***TP53***	***SMAD4***		**Evidence**	**Primary Chr (s)**	
**1**	PDAC	FNA	10	0	1	1	Partial	NO	Too Low	nd
**4**	PDAC	FNB	18	1	1	0	Partial	YES	14, 17q	nd
**7**	PDAC	FNA	43	0	1	1	Complete	YES	6, 9p	G12D
**8**	PDAC	FNA	32	0	1	1	Complete	YES	17	G12R
**9**	PDAC	FNA	59	1	1	1	Complete	YES	12, 19‐22	G12D
**10**	pNET	FNB	88	2(4)	2(4)	2(4)	Complete	YES	1p‐12p (Subclonal)	WT
**11**	PDAC	FNB	nd	2	2	1	Poor	NO	Too Low	Too low
**12**	PDAC	FNB	29	1	1	1	Complete	YES	12‐19,18, 20‐21‐22	G12R
**15**	PDAC	FNB	11	1	1	1	Partial	YES	5q	Too low
**16**	PDAC	FNB	58	1(2)	1(2)	1	Complete	YES	2, 13‐21,	G12V
**17**	PDAC	FNB	74	2	2	2	Complete	YES	3‐4, 4q,	WT
**18**	PDAC	FNB	65	0	1(2)	1(2)	Complete	YES	2‐6, 9, 16	G12C

Note

Bracketed numbers indicate copy number in cases of LOH

Abbreviation: nd, not determined, no additional DNA available for assay.

Figure 1D presents a genome linear plot for PANC17 showing the coverage across each chromosome. Coverage points are coloured according to their bioinformatically determined level; with grey, blue and red dots indicating normal diploid, gains and losses, respectively. Blue gains of 1q, and parts of 1p, 8p, 10p, 12q and 13q and focal red losses of regions of 3p, 3q, 4q, 6q, 11q, 15q and 21q lie at identical coverage levels (3N and 1N) relative to the 2N diploid grey levels, indicative of a homogeneous clonal tumour population. The X and Y coverage levels of this male patient lie slightly lower than the red regions indicative of the true 1N level of the ~ 74% tumour sample (Figure 1B,C). Exemplar coverage across chromosome 6 is presented in the lower panel of Figure 1D, with a homozygous loss of an ~ 450kb region of 6q26 within the *PARK2* gene indicated, with links to pancreatic tumorigenesis.

### 3.2 SVA on higher percentage tumour specimens

**FIGURE 2:**
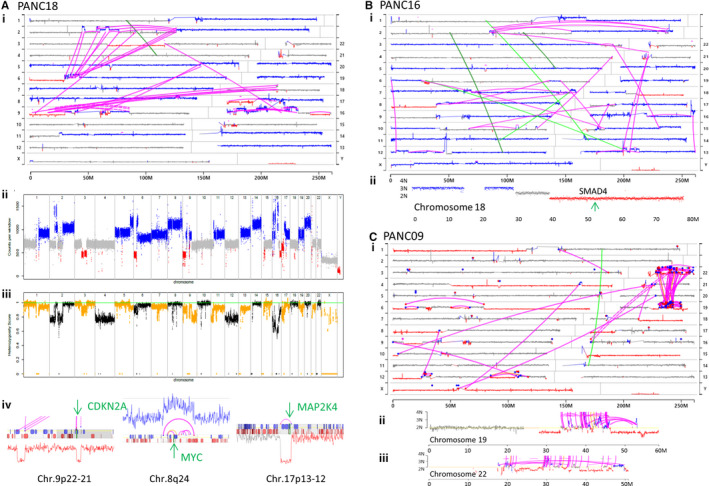
SVA on high percentage tumour specimens. A, Case PANC18. Genome U‐plot of PANC18 (i) presenting chromosomes 1‐12 stacked on the left and 13‐22 on the right, with X and Y at the bottom. Diploid 2N, gains and losses presented by grey, blue and red dots, respectively, for 30kb windows across each chromosome. Lines link discordant mapping DNA junctions where distal regions of chromosomes have been brought together by large genomic rearrangements. High confidence single and balanced junctions are coloured magenta and green, respectively. (ii) Genome linear plot. (iii) Loss of heterozygosity analysis plot. Allelic frequency of SNPs of each chromosome sequentially (x‐axis) with alternating chromosomes coloured orange or black. Upper green line indicates perfect heterozygosity with a score of 1 (y‐axis), and variance indicated by drop in this score. (iv) Coverage across chromosomes 9, 8 and 17, with focal regions for *CDKN2A, MYC* and *MAP2K4*, respectively. B, Genome U‐plot (i) and coverage across chromosome 18, green arrow indicating position of *SMAD4* (ii). C, Genome U‐plot of PANC19 (i) and coverage across chromosomes 19 (ii) and 22 (iii) illustrating connected regions through complex rearrangement

Higher tumour percentages in a tissue specimen allow effective derivations of structural variance, through increased resolution of gains/losses from the normal 2N genome. Eight (67%) tumour specimens predicted tumour percentages > 20% (Figure 1B) and provided detailed SVA by MPseq, 3 of which are exemplified in Figure 2 with additional presented in Figure S1. PANC18 predicted 79% tumour, with significant aneuploidy in a tetraploid genome (Figure 1C). The genome U‐plot in Figure 2Ai provides an alternative visual of the structural variance, including junctions. Magenta lines link discordantly mapping DNA junctions where distal regions of chromosomes have been brought together by large genomic rearrangements. Three complex rearrangement events were clearly identified in this tumour: two inter‐chromosomal shuffling's primarily between chromosomes 2‐6 and 9‐18 and an intra‐chromosomal shuffling on chromosome 16. The genome linear plot revealed the multiple chromosomal copy levels with potential sub clonal populations deviating from the predicted 1N, 2N, 3N and 4N levels (Figure 2Aii). Loss of heterozygosity (LOH) analysis predicted copy‐neutral LOH (cnLOH) for chromosomes 4, 12 and regions of chromosomes 2, 9, 11 and 16 (Figure 2Aiii) highly relevant for potential point mutations within these regions. Homozygous loss of *CDKN2A* on chromosome 9p, gain of *MYC* locus on chromosome 8 through focal chromothriptic rearrangement and a focal deletion on chromosome 17 predicting a homozygous deletion of *MAP2K4* also observed (Figure 2Aiv). Junctions passing algorithmic filters for all cases are presented in Table S3.

PANC16 also predicted a tetraploid genome with 58% tumour content (Figure 1B,C and Figure 2Bi). Deletion of 18q predicts characteristic PDAC loss of *SMAD4* (Figure 2Bii) and cnLOH observed at *CDKN2A* and *TP53* loci (Figure S1). A complex rearrangement was evident linking chromosomes 2, 13 and 21. PANC09 displayed multiple chromosome deletions in a diploid genome, predicting 59% tumour (Figure 2Ci). Deletions on 9p and 17q revealed characteristic *CDKN2A* and *TP53* loss, respectively. A complex rearrangement between chromosomes 19 and 22 impacted many genes with extensive chromosomal shuffling, local gains and losses (Figure 2Cii, Ciii).

The status of *CDKN2A, TP53* and *SMAD4* were clearly resolved in tumours with > 20% tumour (Table 1). For the 7 higher percentage PDAC tumours, 6 (86%) had loss of at least one copy of *CDKN2A*, *TP53* and *SMAD4*, with homozygous loss of *CDKN2A* in 3 (43%) tumours and *SMAD4* in one (14%) tumour. PANC17 predicted wild‐type coverage of each gene. The pNET PANC10 tumour was observed to have a hypodiploid genome, with each of the 3 genes predicting 2 copies. LOH was also predicted in chromosomes 1, 2, 3, 6, 8, 10, 11, 13, 16 and 22 **(**Figure S2 **)**.

### 3.3 Structural variance analysis on low percentage tumour specimens

**FIGURE 3:**
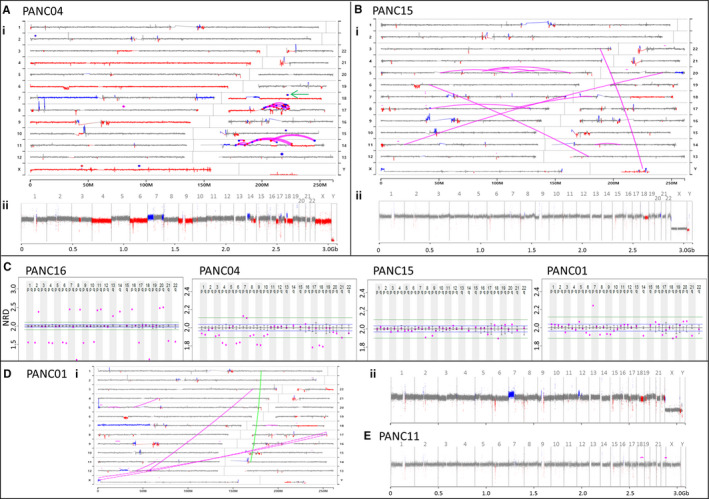
SVA on lower percentage tumour specimens. A, Case PANC04. Genome U‐plot (i) and linear plot (ii) with copy gains and losses indicated in blue and red, respectively. Junctions presented in magenta and SMAD4 spanning deletion on chromosome 18q indicated by green arrow. B, Case PANC15. Genome U‐plot (i) and linear plot (ii). C, Aneuploidy plots of PANC16, PANC‐04, PANC‐15 and PANC‐01. NRD of each chromosomal arm (magenta dots) relative to diploid 2N level. Error bars indicate variance across normal controls for chromosomal arms, with standard variance levels indicated by blue lines. Green lines indicate ~ 10% algorithmic calling limits. D, Case PANC01 genome U‐plot (i) and linear plot (ii). E, Linear genome plot of PANC11

Four specimens had tumour percentages of < 20%, challenging the SVA (Figure 3). Although PANC04 predicted 18% tumour, chromosomal copy changes were clearly resolved (Figure 3Ai), but with reduced resolution from the grey 2N levels (Figure 3Aii). While junctions clearly identify two complex rearrangements on chromosomes 14 and 17, lower numbers of supporting fragments drop a subset of junctions below bioinformatics thresholds at these lower tumour percentages. Single loss of *CDKN2A* and *TP53* are predicted, with homozygous loss of *SMAD4* through a secondary focal spanning deletion on chromosome 18q (Figure 3Ai green arrow). Copy changes were more difficult to resolve in PANC15 (11% tumour content), but a chromothriptic event is clearly observed on chromosome 5 (Figure 3Bi, Bii).

In low percentage tumours, copy number gains/losses can be better resolved by normalized read depths (NRS) of whole chromosome arms, rather than 30kb windows (Figure 3C and Figure S3 **)**. For PANC16, 1N and 3N NRD calls appear primarily at 1.5 (losses) and 2.5 (gains), respectively, consistent with the 58% tumour. While the majority of NRD levels are resolved for PANC04 (18% tumour), for PANC15 and PANC01 (~10% tumours) most NRDs lie below the ~ 10% algorithmic calling thresholds (green lines). However, many calls are still resolved from the expected variance levels across normal controls (blue lines). In both cases, 9p (*CDKN2A*), 17p (*TP53*) and 18q (*SMAD4*) losses are indicated consistent with PDAC (Figure 3C). An additional focal deletion of 9p on PANC01 by junctions infer a homozygous loss of *CDKN2A* (Figure 3Di). While PANC11 had undetermined tumour content (Figure 3E), a junction predicted loss of *SMAD4* (Table 1).

### 3.4 *KRAS* mutation detection

*KRAS* G12 mutations were detected in 6 of 8 specimens containing > 20% tumour from independent Sanger sequencing. (Table 1) No *KRAS* mutation was seen for PANC10, consistent with a pNET. PDAC tumour PANC17 had satisfactory tumour content (74%) but presented with wild‐type *KRAS* G12/G13, in addition to no loss of *CDKN2A, TP53* or *SMAD4*. Sanger sequencing failed to resolve the presence of G12/G13 mutation in two specimens with lower tumour contents, where sufficient DNA was available.

### 3.5 Transcriptomic profiling of EUS specimens

**FIGURE 4:**
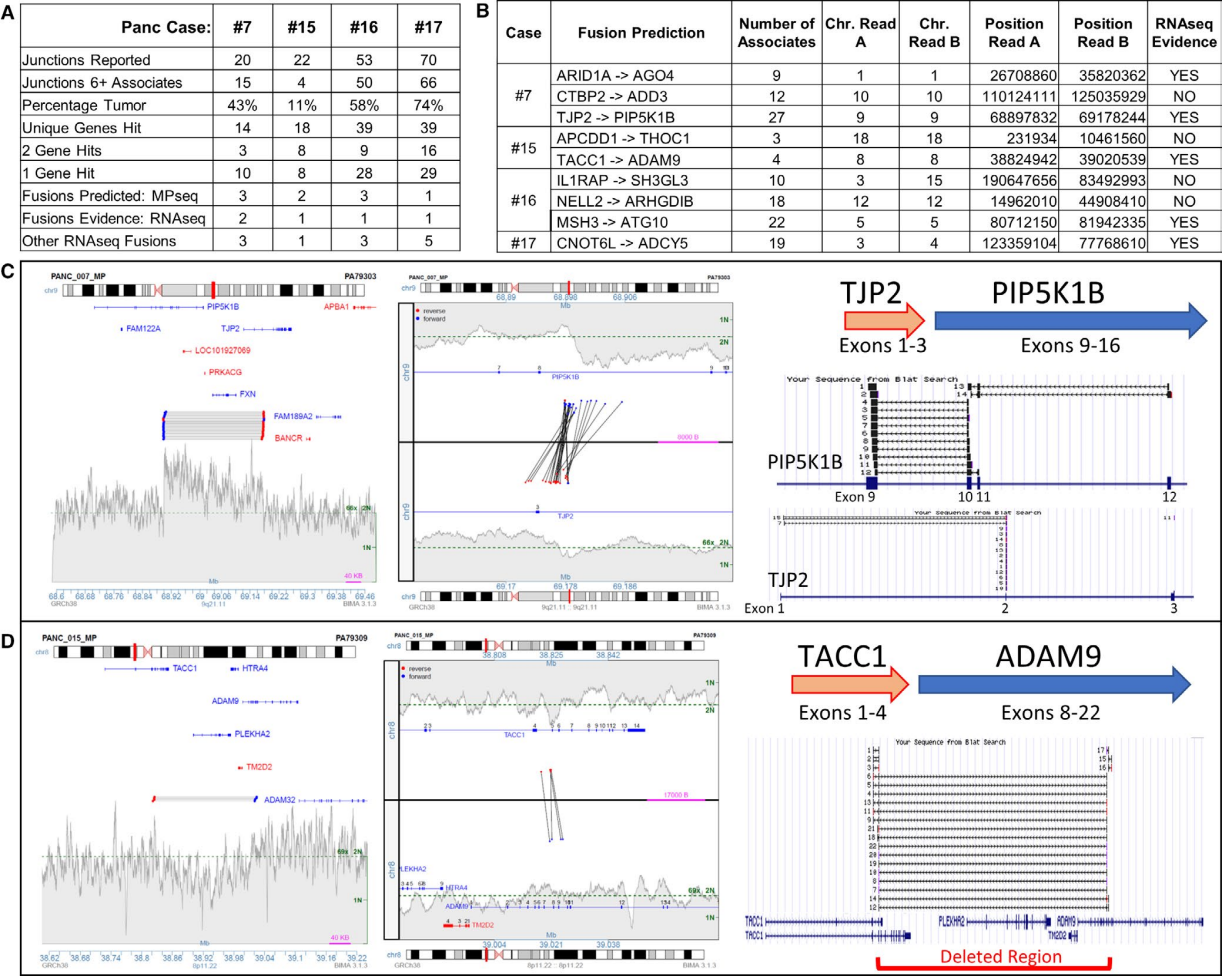
Fusion detection in PDAC A, Numbers of junctions reported in DNA by MPseq and in RNA for cases additionally profiled by RNAseq. B, Description of fusion transcripts detected in MPseq and RNAseq. C and D, Illustrations of TJP2‐PIPK1B and TACC1‐ADAM9 fusions in PANC07 and PANC15, respectively. Left images illustrate region plots of the supporting fragments spanning the junctions and the genes present at these loci. Central images illustrate the junction plots of supporting fragments spanning the two breakpoints (upper and lower panels). The lines linking the upper and lower panels link tfigurehe breakpoint positions relative to the hit genes. Red and blue dots indicate reads mapping to the positive or negative strands, respectively. The shaded grey areas indicate the coverage across each region.^27^ The right images illustrate the mapping of RNAseq supporting fragments using NCBI BLAT and the resultant fusion structures. While the supporting fragments predicting a fusion from MPseq data map to the DNA breakpoint junction, which is often intronic, in RNAseq the fusion supporting fragments map precisely to exons

RNAseq on a subset of cases where sufficient RNA was available (PANC07, PANC15, PANC16, PANC17) evaluated fusions predicted in MPseq. The numbers of detected junctions in these cases ranged from 20 to 70 (Figure 4A). Many junctions had ≥ 6 supporting fragments; however, this confidence level dropped in the lower percentage tumours. A subset of junctions hit two genes in the correct orientation for expression of fusion peptides. Of the 10 gene fusions predicted by MPseq, RNAseq evidence supported five (Figure 4A,B). While the expression‐driving 5’genes in the detected fusions (ARID1A, TJP2, TACC1, MSH3, CNOT6L) were each significantly expressed (RPKM:3.6‐58.4), expressions were lower in the non‐detected group (RPKM:0.6‐2.4) (Table S4). The structure and evidence for the TJP2‐PIP5K1B and TACC1‐ADAM9 fusions are presented in Figure 4C,D, respectively.

### 3.6 Potential clinically relevant variations from SVA

Table 2 lists a summary of a subset of potential relevant clinical genes impacted in the PDAC patients.

**TABLE 2:** Potential clinically significant somatic variants

**Case**	**Gene**	**CSG**	**Call**	**Modification**	**Significance**	**Potential Action**	**Ref's**
**1**	KRAS	OG	G	Undetermined mutation	Potential gain of mutant allele	Prognostic Marker, KRAS trials	^27^
	CRLF2	OG	G + F	CYB1‐CRLF2	JAK/STAT Pathway initiator of PDAC tumours	Therapeutic Target	^28^
	CDH10	TSG	T	Truncation Loss	Linked to PDAC carcinogenesis	Prognostic Marker	^29^
**4**	NLK	TSG	F	NSF‐NLK	Ser/Thr Kinase involved in WNT/MAPK/ERK pathway	Therapeutic Target	^31^
	NPAS3	TSG	F	MIPOL1‐NPAS3	Fusions reported. Negative prognostic marker in astrocytomas	Prognostic Marker	^32^
	MAPT		L	Potential homozygous loss	Poor prognosis &decreased response to taxane‐based therapies in several cancers	Prognostic Marker	^32^
**7**	ARID1A	TSG	F, L	ARID1A‐AGO4	SWI/SNF component restrains PDAC formation. Loss increases MYC. Potential for PARP Inhibitors. + Radiotherapy	Therapeutic Target	^33^
	AGO4		F	ARID1A‐AGO4	Argonaut proteins overexpressed in colon cancer; promote Met's	Therapeutic Target	^21^
	PIP5K1B		F	TJP2‐PIP5K1B	PIPK regulation. Organization actin filament through RAC1. WNT signalling.	Therapeutic Target	^22^
	RASA1		G	High Expression	RET and ERK signalling. Driver of PDAC progression.	Therapeutic Target	^34^
**8**	MAP3K13	TSG	F	SENP2‐MAP3K13	Positive regulator of MYC to promote tumour development	Therapeutic Target	^35^
	PAK4		G	Copy level gains	Novel PAK4 allosteric modulators (PAM) on a panel of PDAC	Therapeutic Target	^36^
	GATA6		G	Copy level gains	Transcriptional activator regulates EMT and MET's. Expression distinguishes classical/basal‐like subtypes in PDAC	Diagnostic/Prognostic	^37^
	MAP4K1		G	Copy level gains	Functions in JNK/MYC signalling cascades	Therapeutic Target	^38^
**9**	MAPK1	OG	F	RTN2‐MAPK1	ERK Inh; Ulixertinib promise in recent clinical trials. KRAS G12D mutation specifically activates ERK2 and invasion in PDAC	Therapeutic Target	^39^
	MORC2		F	COMT‐MORC2	Regulates DNA damage responses; PARP1‐dependent pathway	Therapeutic Target	^40^
	SSH1		F	BTBD11‐SSH1	Sennoside A novel inhibitor of SSH family proteins	Therapeutic Target	^41^
	DGCR8	OG	T	DGCR8‐no_gene (ex1‐12)	Terminal truncation of oncogene. Chr. Del'n syndrome. Metastasis	Therapeutic Target	^42^
	CLTCL1	TSG	T	Truncation Loss	Early driver of progression in breast ductal carcinoma. NOTCH signalling.	Therapeutic Target	^43^
**12**	CNTN1		F	NELL2‐CNTN1	Dysregulate the NOTCH pathway, Tarextumab tested in PDAC	Therapeutic Target	^44^
	SND1	OG	bF	CLVS1‐SND1	Involved in tumour proliferation, inflammation, invasion, and metastasis	Therapeutic Target	^45^
	CACNA1G		F	ABCC3‐CACNA1G	Voltage‐gated calcium and potassium channels; implications for cell proliferation and apoptosis	Prognostics Therapeutic Target	^46^
	CACNA1B		F	TUBBP5‐CACNA1B			
	KCNMA1		F	STN1‐KCNMA1			
	CDH4		F	ASXL3‐CDH4	Ca‐dep cell‐cell adhesion glycoprotein cadherin 4; MET association	Prognostic Marker	^47^
**15**	ADAM9		F	TACC1‐ADAM9	Potentially affects cisplatin‐based therapies	Therapeutic target	^23^
	THOC1		F	APCDD1‐THOC1	Aggressive phenotype and poor prognosis in colorectal cancer	Therapeutic target	^48^
	YES1		G	Gain Region	High expression linked carcinogenesis and metastasis	Therapeutic Target	^49^
**16**	SH3GL3		F	IL1RAP‐SH3GL3	Cell migration/invasion. Chemo‐resistance. FAK/PI3K pathways	Therapeutic target	^50^
	ARHGDIB		F	NELL2‐ARHGDIB	Rho family signalling. Proliferating and cytoskeletal organization.	Therapeutic Target	^51^
	ATG10		F	MSH3‐ATG10	Increased expression associated with lymphovascular invasion and LN metastasis	Prognostic Marker	^27^
	AMIGO2		G	High Expression	Potential targeting with BET Inhibitors.	Therapeutic target	^52^
**17**	LRP1B	TSG	L	Truncation, Loss	Deletion linked to poor outcome and resistance to therapy.	Prognostic Marker	^53^
	TERT	TSG, OG	G	High Expression	Role in cell growth; tumorogenesis and progression.	Therapeutic target	^54^
	FAT1	TSG	T	Truncation Loss	Inhibitor of proliferation and metastasis. Loss promotes resistance to CDK inhibitors.	Prognostic Marker, Targetable	^55^
	RET	OG	G	High Expression	Up‐regulation Induces Perineurial Invasion of Pancreatic Adenocarcinoma	Prognostic Marker, Targetable	^56^
	DAXX	TSG, OG	G	High Expression	Expression correlates with tumorigenesis, disease progression, treatment resistance.	Prognostic Marker	^57^
**18**	REL	OG	F	LINC01793‐REL	Involved in apoptosis, inflammation, the immune response, and oncogenic processes.	Therapeutic target	^58^
	LRP1B	TSG	L	Intragenic deletion	Deletion linked to poor outcome and resistance to therapy.	Prognostic Marker	^54^
	MSH2	TSG	F	MSH2‐ANKS1A	MSI implications; Immunotherapy or PARP inhibitors.	Therapeutic target	^59^
	ANKS1A		F	MSH2‐ANKS1A	Regulator of multiple signalling pathways; EGF family. Antagonists. SRC family kinase target.	Therapeutic target	^59^

Abbreviation: bF, Balanced Fusion; CCG, Cosmic Census Genes; F, Fusion; G, Gain; L, Loss; OG, Oncogene; T, Truncation; TSG, Tumour Suppressor Gene.

Gene fusions result in change of function expression of chimeric proteins. Of the productive fusions reported in Figure 4B; AGO4 and PIP5K1B are both involved in RET and PI3K signalling ^21^, ^22^ and expression of ADAM9 is a prognostic factor for PDAC vascular invasion and potentially targetable with novel antibody‐drug conjugates.^23^ Increased expression of *ATG10* in colorectal cancer is also associated with invasion and metastasis.^24^ Other fusions in cases without RNAseq confirmatory evidence were also clinically significant. In PANC09, the *MAPK1* (*ERK2*) oncogene fusion would be of high interest for therapeutic targeting, together with *MORC2* and *SSH1* fusions. PANC12 fusions included the *SND1* oncogene and contactin 1 (*CNTN1*), but also 3 different ion channels and the calcium‐dependent cadherin 4 (*CDH4*) gene. Other potential clinically relevant fusions included *CRLF2* in PANC01, *NLK* in PANC04, *MAP3K13* in PANC08 and the *REL* oncogene, *MSH2* and *ANKS1A* in PANC18. Additional tumour suppressor genes (TSGs) were also impacted by deletion or truncating events, including Cadherin 10 (*CDH10*) in PANC01, *CLTCL1* in PANC09, *FAT1* in PANC17 and *LRP1B* in both PANC17 and 18. Conversely, the oncogene *DGCR8* is truncated of just the terminal exons in PANC09 which could provide oncogenic function. *TERT, RET* and *DAXX* oncogenes were each observed to be gained and highly expressed in PANC17, which could be considered major targetable event in this cancer. Other examples of significant gains were observed for *KRAS* in PANC01, *RASA1* in PANC07, *PAK4, GATA6* and *MAP4K1* in PANC08, *YES1* in PANC15.

The potentially relevant clinical genes listed in Table 2 were additionally evaluated for their relevance in previously reported PDAC genomics studies (Table S5). Sixteen of the 40 gene (40%) were similarly hit by DNA junctions in previously reported MPseq of 68 independent PDAC tumours.^9^, ^25^ Somatic variants were reported in all 40 of the genes from genomic sequencing of 324 PDAC tumours listed in public data from TCGA Firehouse Legacy and TCGA PanCancer Atlas studies on the cBioPortal.^26^ Somatic variation was reported in 16 (40%) and 7 (17%) of the genes at frequencies of > 3% and > 5% PDAC tumours, respectively, consistent with the known heterogeneity of pancreatic tumours (Table S5). Eight (20%) of the genes (*ARID1A, CDH10, FAT1, GARA6, KRAS, LRP1B, PAK4* and *YES1*) were hit by junctions and present in > 3% PDAC cases in both TCGA datasets.

### 3.7 Detection of novel peptide transcripts in PDAC from DNA fusions

**FIGURE 5:**
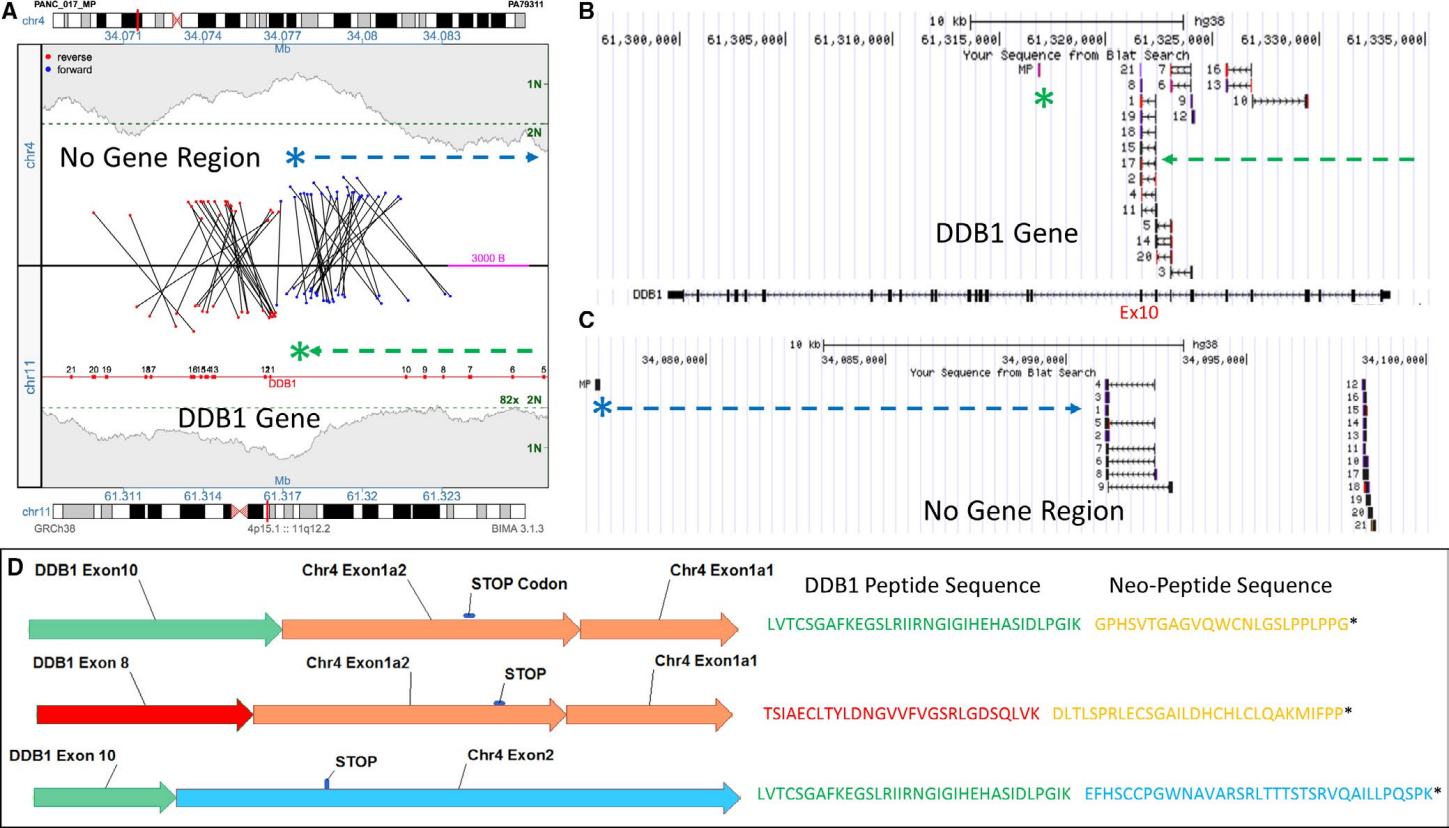
Neo‐Peptides from DDB1 truncation. A, Junction plot illustrating the supporting fragments spanning the upper non‐genic region of chr.4 and the lower DDB1 gene on chr.11. The green asterisk and line illustrate the breakpoint and direction of the truncating DDB1 gene. The blue asterisk and line illustrate the breakpoint and direction of sequence on chr.4. B and C, NCBI BLAT of reads from supporting fragments mapping to the DDB1 gene on chr.11 and non‐genic region on chr.4. D, Structure of readthrough fusion transcripts and the resultant neo‐peptide amino acid sequences

In addition to gene‐gene fusions, several RNAseq fusion transcripts were reported where MPseq predicted gene truncations with no aligned 3’fusion gene. Figure 5A illustrates a *DDB1* junction in PANC17 where DDB1 transcripts would be predicted to terminate at exon 10. However, transcripts extending from *DDB1* exon 10 into chromosome 4p15.1 were observed. Reads on 11q12.2 map predominantly to *DDB1* exons 8, 9 and 10, with some also covering splicing to exons 4, 5, 6 and 7. (Figure 5B) The 4p15.1 reads also mapped in exon like fashion in non‐genic areas of NCBI Refseq or Ensembl. (Figure 5C) Thus, the RNA splicing machinery is extending from DDB1 exon 10 and linking transcripts with novel exonic structures. Three main transcripts were predicted. Two mapped from exon 8 or 10 of *DDB1* to two similarly spliced neo‐exon 4p15.1 location (Figure 5D, Chr4 Exon1a1 and 1a2) and a third spliced exon 10 of *DDB1* to a more distal region on 4p15.1 (Chr4 Exon 2). Three distinct novel peptides were predicted adding 24, 30 and 35 amino acids before reaching a stop codon. Additional novel peptide transcripts were predicted in the four PDAC tumours (Table S6).

## 4 DISCUSSION

Rearrangement driven ‘change of function’ effects on genes arise in tumours by several mechanisms, including gene fusions, truncations, haploinsufficiency, homozygous deletions, gains / amplifications, and modified promoter and enhancer functions. In this study, we demonstrate the feasibility of using treatment‐naïve PDAC tumour biopsy specimens for whole genome‐based mate pair sequencing to define clinically relevant structural variations that may have prognostic and therapeutic potential. All specimens (mean tumour percentage of 41%) provided good quality DNA for MPseq using either a 22G FNA or FNB needle to acquire diagnostic material. Higher percentage tumours (> 20%) enabled high‐quality SVA, unveiling a complete picture of the somatic variants present. The status of the commonly deleted *TP53*, *CDKN2A* and *SMAD4* genes was precisely mapped in these tumours at levels consistent with other studies.^3^, ^4^, ^5^, ^6^, ^7^, ^8^, ^9^ Although lower percentage tumours (< 20%) challenged SVA, they provided informative evidence of structural variance in most specimens.

Complex rearrangement events were observed in 83% of specimens with just two specimens failing to reveal any complex rearrangements due to lower tumour cellularity resulting in lower tumour sequencing depths and reduced numbers of associate reads for somatic DNA junctions. The pattern of these complex events was quite unique for each case; with minimal commonality in chromosomal regions hit. Chromothryptic/chromoanasynthetic events involving single chromosomes were evident in 5 tumours frequently involving both gains and losses adjacent to junctions. Conversely, many of the cases involved chromoplectic like events with multiple linked chromosomes hit, ranging from 3 to 12 separate chromosomes, with again regions of gain and loss adjacent to junctions. The pNET tumour contained a chromothriptic event linking two small regions on chromosome 1p and 12p. However, reduced numbers of supporting fragments spanning these junctions predict its presence in a sub‐population of tumour cells (~10%).

PDAC tumours are classically challenging to define targetable variants. Clinical genomic evaluation of such tumours currently focusses on limited gene panels; however, beyond the common *KRAS, TP53, SMAD4* and *p16* variants, additional genes on these panels are infrequently mutated. The heterogeneity of PDAC tumours, therefore, demands a more extensive genomic evaluation. Point mutations describe just one dimension of somatic variation, with structural and epigenetic variations, together with transcriptome, required to comprehensively evaluate altered gene functions. Many genes are insensitive to haploinsufficiency, with a remaining wild‐type copy frequently preventing oncogenic progression, and so detailed knowledge of both alleles of a gene is necessary to determine the impact of the alteration. SVA in this study was able to effectively identify chimeric fusion proteins, change of function truncations, gains and losses of key genes linked to tumour progression, many with potential clinical significance. The proposed variations described are speculative and require additional validation to infer relevance to PDAC treatment. The clinical implementation of therapeutic strategies to target genomic variants is also, however, currently lagging well behind our abilities to detect these variants. This stems from several legitimate practical issues including an understandable reluctance to move from mainline treatment strategies, but primarily from limited access to FDA‐approved drugs and a lack of data indicating their clinical efficacy. This is further exasperated by single targeting therapies often being insufficient to reduce tumour burden and combination therapies hitting multiple pathways required, further complicating clinical trials. Nevertheless, targeted therapies are expected to rapidly increase as these limitations are reduced, and therefore, clinical genomics testing needs be ready to provide the essential data, at the earliest interventional time, to direct the best clinical therapeutic strategies. A review of the EUS tumour specimens investigated in this study provides a wide repertoire of potentially clinically relevant somatic variants, emphasizing the heterogeneity in these tumours. Nevertheless, each variant can provide subtle clues to define novel future therapies or inform of potential negative effectors of proposed treatments. Many variants also provide prognostic inference, while less significant in commonly aggressive PDACs, they could be informative in early treatment‐naïve tumour specimens.

In addition to fusion peptide validation, RNAseq data also revealed the presence of novel tumour specific transcripts generated from gene truncating junctions with readthrough expression into novel exon motifs in non‐genic regions. This novel class of readthrough peptides, in addition to change of function effects, would be foreign to the patient's immune surveillance and potentially be presented by MHCII molecules to circulating T cells. Immune checkpoint blockades play a major role in preventing these antigen recognition pathways; these novel peptides could, however, benefit patient response to immunotherapy treatments. Response to immunotherapy in PDAC to date has been rare, but recent reporting presents optimism in future applications.^2^ Early derivation of potential responders to immunotherapy will benefit patients, for which the described techniques in the presented study will be invaluable tools to enable these screening guides.

Treatment breakthroughs on a subset of PDAC patients are progressively being achieved through advances in genomic and molecular profiling of patient tumours.^60^ Further combinatorial efforts of genomic profiling with real‐time 3D micro‐cancer model systems ^61^ will further accelerate these studies in evaluating the efficacy of targeting drugs against common somatic variants and anticipate the resistance mechanisms that eventually lead to treatment failure, while proactively designing combinatory targeting strategies. A recent multi‐platform molecular analysis of 150 surgically resected PDAC specimens reported stratification of cases into prognostic subtypes and potential therapeutic opportunities, described as a roadmap for precision medicine and genotype‐directed clinical trials.^62^ Although this report insightfully detailed the fate of clinically relevant genes through integrated computation of whole exome, RNAseq, methylation and proteomics antibody array, much of the structural variance reported in this study would have been overlooked in their theragnostic evaluations.

In conclusion, we demonstrate the suitability of EUS‐guided solid pancreas tumour biopsies from a broad spectrum of disease stages to the whole genome‐based sequencing technique termed MPseq, with integrated transcriptomics analysis on a subset of available specimens. The prevalence and pattern of structural variance, including chromoanagenesis, were identified with possible prognostic biomarkers and therapeutic targets in the treatment‐naïve pancreas cancer EUS biopsy specimens. SVA of all samples with tumour > 20% provided detailed genome‐wide impact on clinically relevant genes and their expression. Although SVA was challenged with lower percentage tumours down to 10%, the results were highly informative and could provide clinically relevant profiles of the tumours and their genomic instability. Large numbers of junctions were predicted from structural rearrangements, with chromoanagenesis detected in all high percentage tumour samples. Multiple clinically significant genes were identified as impacted in each case with potential for therapeutic targeting or immune based therapies, emphasizing the importance of SVA in clinical testing. In conclusion, EUS‐guided biopsies can aid precision medicine‐directed therapy for pancreas cancer patients as an invaluable frontline resource of tissue for early evaluation of the genomic landscape of somatic variation, which drive these challenging tumours.

## CONFLICT OF INTEREST

The authors do not have any financial interests related to the technology presented. George Vasmatzis wishes to disclose that he is the owner of WholeGenome LLC.

## AUTHOR CONTRIBUTION

**Stephen J Murphy:** Conceptualization (equal); Data curation (lead); Formal analysis (supporting); Funding acquisition (supporting); Investigation (supporting); Methodology (lead); Project administration (supporting); Supervision (supporting); Visualization (supporting); Writing‐original draft (lead); Writing‐review & editing (supporting). **Michael J Levy:** Conceptualization (supporting); Methodology (supporting); Writing‐review & editing (supporting). **James Smadbeck:** Formal analysis (lead); Software (lead); Writing‐original draft (supporting). **Giannoula Karagouga:** Data curation (supporting); Methodology (supporting); Writing‐review & editing (supporting). **Alexa Mccune:** Data curation (supporting); Methodology (supporting); Writing‐review & editing (supporting). **Faye Harris:** Data curation (supporting); Methodology (supporting); Writing‐review & editing (supporting). **Julia Udell:** Formal analysis (supporting); Software (supporting); Writing‐review & editing (supporting). **Sarah Johnson:** Data curation (supporting); Formal analysis (supporting); Software (supporting); Visualization (lead); Writing‐review & editing (supporting). **Sarah Kerr:** Conceptualization (supporting); Data curation (supporting); Formal analysis (supporting); Methodology (supporting); Writing‐review & editing (supporting). **John C. Cheville:** Methodology (supporting); Resources (supporting); Writing‐review & editing (supporting). **Benjamine Kipp:** Investigation (supporting); Methodology (supporting); Writing‐review & editing (supporting). **George Vasmatzis:** Conceptualization (supporting); Formal analysis (supporting); Funding acquisition (supporting); Investigation (supporting); Methodology (supporting); Project administration (supporting); Resources (lead); Software (lead); Supervision (supporting); Visualization (supporting); Writing‐original draft (supporting); Writing‐review & editing (supporting). **Ferga Gleeson:** Conceptualization (equal); Data curation (supporting); Funding acquisition (lead); Investigation (lead); Methodology (supporting); Project administration (lead); Resources (lead); Supervision (supporting); Writing‐original draft (lead); Writing‐review & editing (lead).

## Supporting information

Table S1‐S6

Figure S1‐S3

## Data Availability

The data that support the findings of this study are available from the corresponding author upon reasonable request.
