# Supplementary material for: Theragnostic chromosomal rearrangements in treatment‐naive pancreatic ductal adenocarcinomas obtained via endoscopic ultrasound
Source: J Cell Mol Med. 2021 Mar 11;25(8):4110–23. doi: 10.1111/jcmm.16381 (PMC8051743; doi:10.1111/jcmm.16381)
Supplement: Supplementary file 2 — Figure S1‐S3 [file JCMM-25-4110-s001.pdf]

Supplemental Figure 1. Additional Genome and Linear Plots.

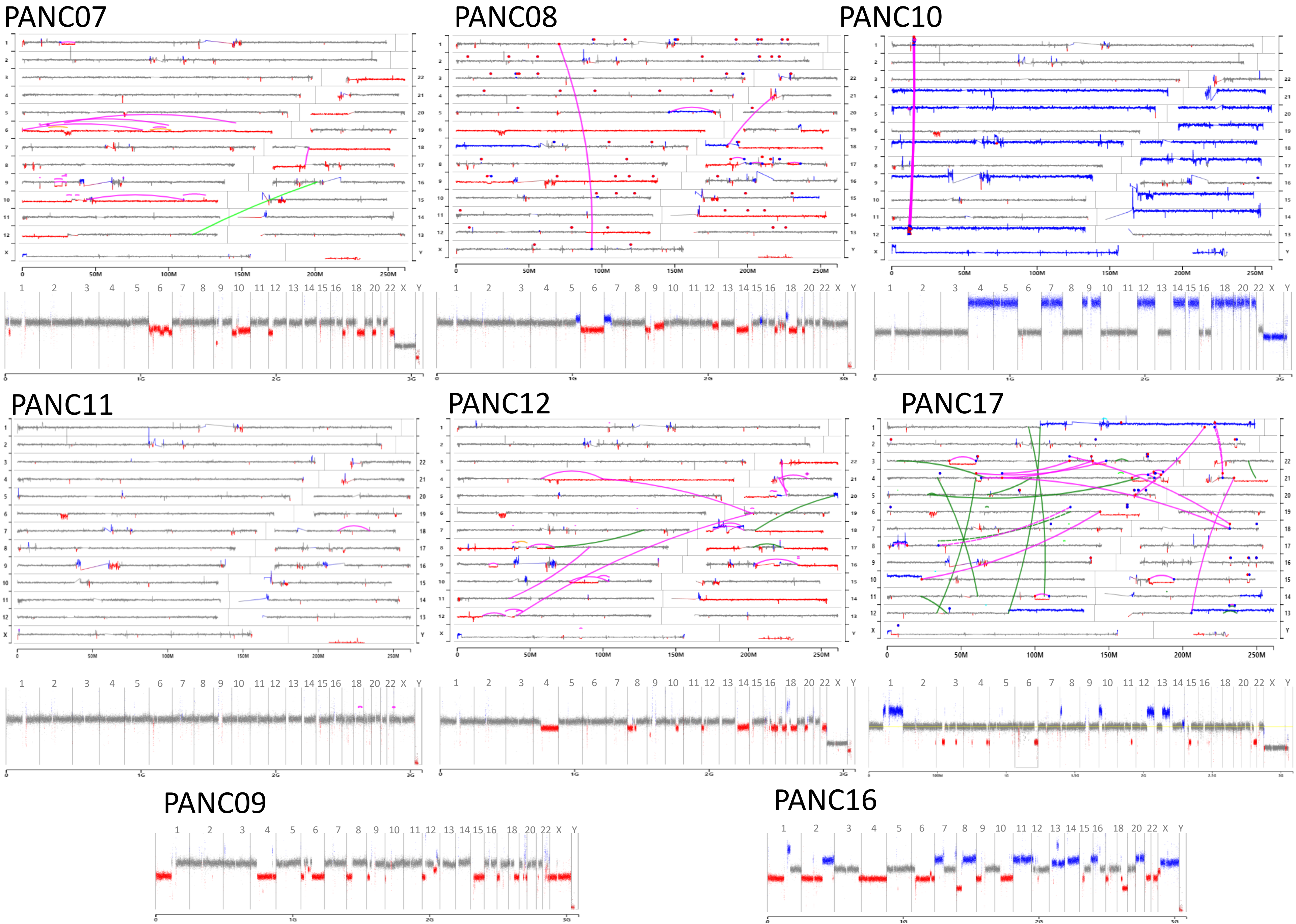

Supplemental Figure 2. Loss of Heterozygosity (LOH) Plots

## PANC10 pNET

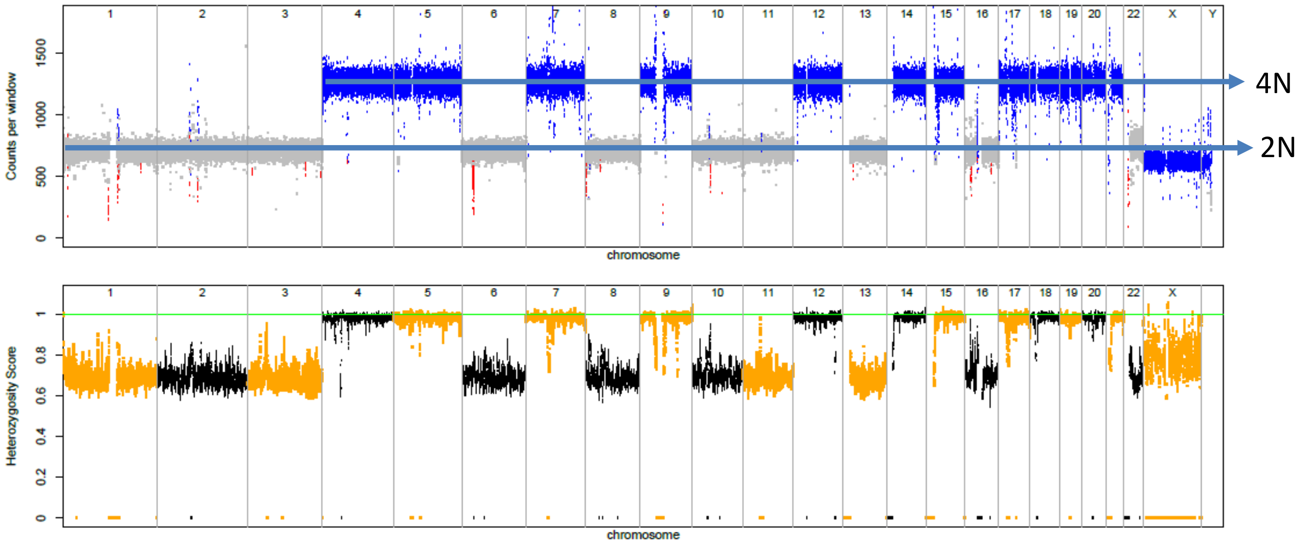


## PANC16 PDAC

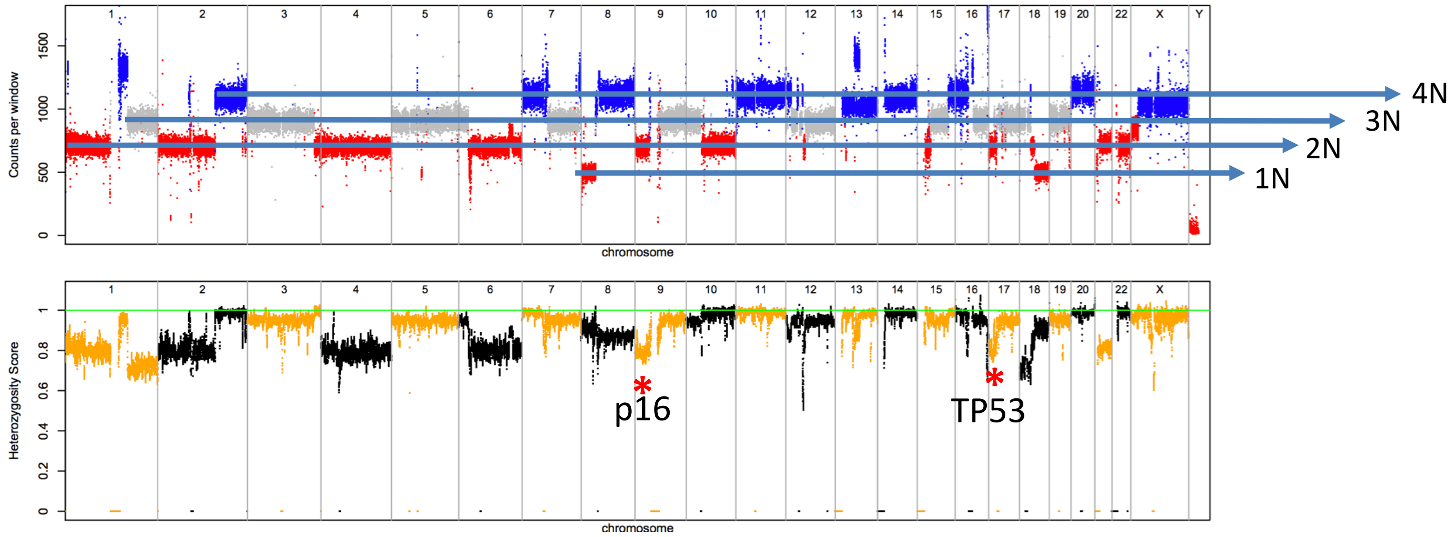

Supplemental Figure 3. Aneuploidy plots of normalized read depths ranked in order of decreasing tumor %.

PANC01 23%

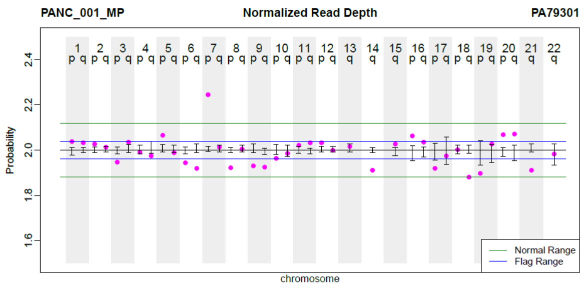


PANC04 18%

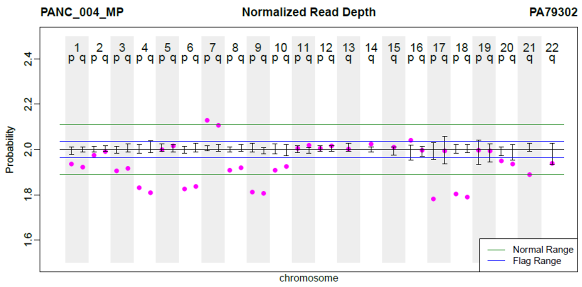


PANC07 43%

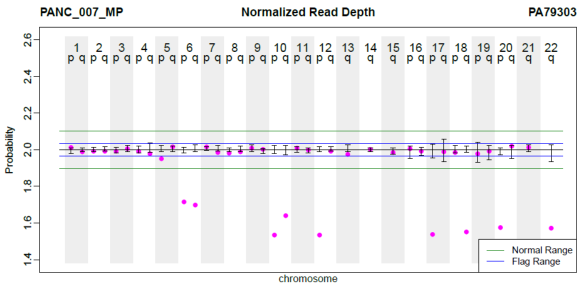


PANC08 32%

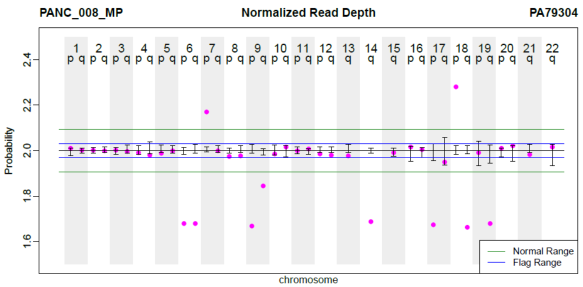


PANC09 59%

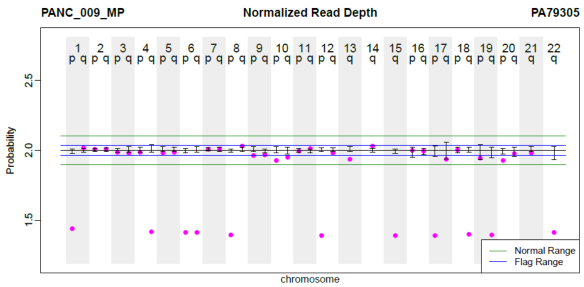


PANC10 87%

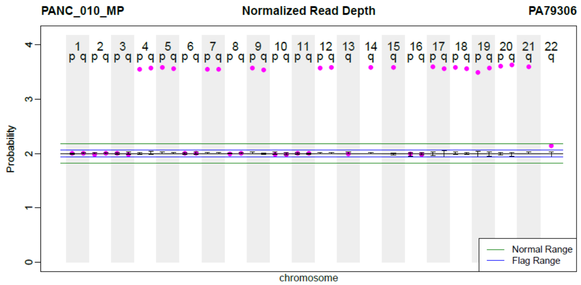


PANC11 nd%

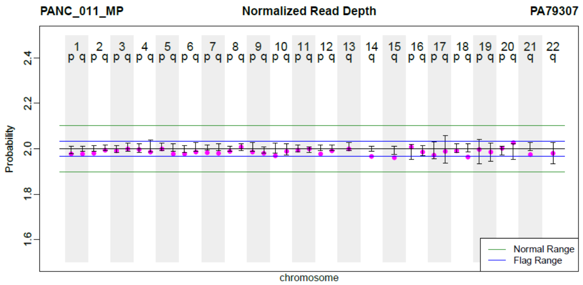


PANC12 29%

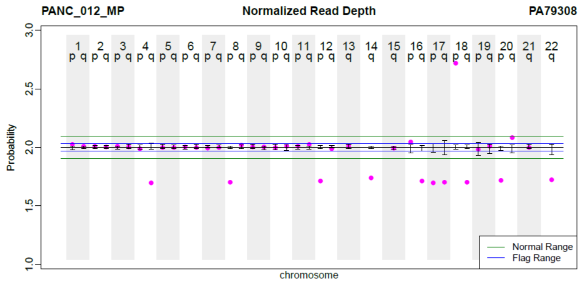


PANC15 11%

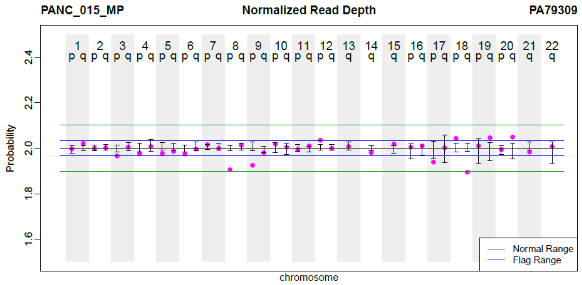


PANC16 58%

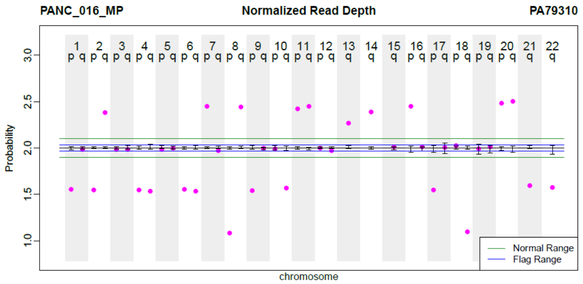


PANC17 74%

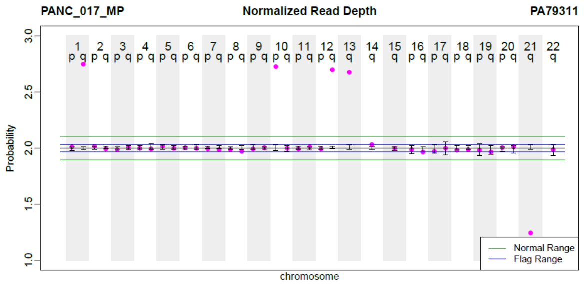


PANC18 65%

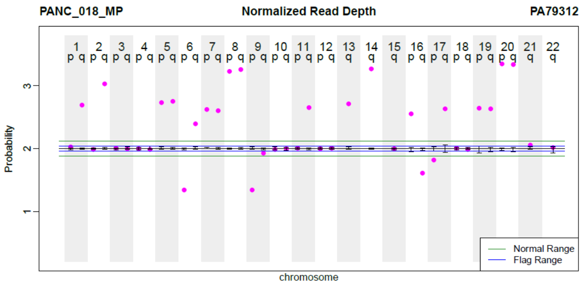

## Supplemental Figure Legends.

**Supplemental Figure 1.** Additional Genome and Linear Plots

Genome stack and linear plots for additional cases. Genome stack plots showing chromosomes 1-12 stacked on the left and 13-22 on the right, with X and Y at the bottom. Diploid 2N, gains and losses presented by grey, blue and red dots, respectively, for 30kb windows across each chromosome. Lines link discordant mapping DNA junctions where distal regions of chromosomes have been brought together by large genomic rearrangements. High confidence single and balanced junctions are colored magenta and green, respectively. Genome linear plot for case show frequency coverage in 30kb window sizes across whole genome presented horizontally for sequential chromosomes 1-22, X and Y. Normal diploid 2N level presented by grey dots. Gains and losses indicated by blue and red dots, respectively.

**Supplemental Figure 2.** Loss of Heterozygosity Plot for PANC10 PDAC and PANC16 pNET

LOH plot of all chromosomes aligned below genome linear plots. Upper linear plots have predicted N levels indicated with horizontal arrows. Chromosome allele coverage of each chromosome sequentially (1-22, X and Y; x-axis) with alternating chromosomes colored orange or black. Upper green line indicates expected 2N coverage with heterozygosity score of 1 (y-axis), with both gains and loss variance indicated by drop in this score.

**Supplemental Figure 3.** Aneuploidy Plots

Aneuploidy plots and tumor percentages presented for all cases. Plots show probability scores of chromosomal arm coverage levels (magenta dots), relative to diploid 2N level. Error bars indicate variance across normal controls for chromosomal arms, with standard variance levels indicated by blue lines. Green lines indicate 10% algorithmic calling limits.
